# Prevalence and Determinants of the Gender Differentials Risk Factors of Child Deaths in Bangladesh: Evidence from the Bangladesh Demographic and Health Survey, 2011

**DOI:** 10.1371/journal.pntd.0003616

**Published:** 2015-03-06

**Authors:** Md. Mosharaf Hossain, Kulanthayan K. C. Mani, Md. Rafiqul Islam

**Affiliations:** 1 Department of Community Health, Faculty of Medicine and Health Science, Universiti Putra Malaysia, Serdang, Selangor, Malaysia; 2 Department of Population Science & HRD, University of Rajshahi, Rajshahi, Bangladesh; Emory University, UNITED STATES

## Abstract

**Background:**

The number of child deaths is a potential indicator to assess the health condition of a country, and represents a major health challenge in Bangladesh. Although the country has performed exceptionally well in decreasing the mortality rate among children under five over the last few decades, mortality still remains relatively high. The main objective of this study is to identify the prevalence and determinants of the risk factors of child mortality in Bangladesh.

**Methods:**

The data were based on a cross-sectional study collected from the Bangladesh Demographic and Health Survey (BDHS), 2011. The women participants numbered 16,025 from seven divisions of Bangladesh – Rajshahi, Dhaka, Chittagong, Barisal, Khulna, Rangpur and Sylhet. The 𝟀^2^ test and logistic regression model were applied to determine the prevalence and factors associated with child deaths in Bangladesh.

**Results:**

In 2011, the prevalence of child deaths in Bangladesh for boys and girls was 13.0% and 11.6%, respectively. The results showed that birth interval and birth order were the most important factors associated with child death risks; mothers’ education and socioeconomic status were also significant (males and females). The results also indicated that a higher birth order (7 & more) of child (OR=21.421 & 95%CI=16.879-27.186) with a short birth interval ≤ 2 years was more risky for child mortality, and lower birth order with longer birth interval >2 were significantly associated with child deaths. Other risk factors that affected child deaths in Bangladesh included young mothers of less than 25 years (mothers’ median age (26-36 years): OR=0.670, 95%CI=0.551-0.815), women without education compared to those with secondary and higher education (OR =0 .711 & .628, 95%CI=0.606-0.833 & 0.437-0.903), mothers who perceived their child body size to be larger than average and small size (OR= 1.525 & 1.068, 95%CI=1.221-1.905 & 0.913-1.249), and mothers who delivered their child by non-caesarean (OR= 1.687, 95%CI=1.253-2.272).

**Conclusion:**

Community-based educational programs or awareness programs are required to reduce the child death in Bangladesh, especially for younger women should be increase the birth interval and decrease the birth order. The government should apply the strategies to enhance the socioeconomic conditions, especially in rural areas, increase the awareness program through media and expand schooling, particularly for girls.

## Author Summary

Children are a significant asset of a country. Child deaths are an important way to determine the health sector development. The effectiveness of the interventions is required to prevent child deaths. The purpose of this study is to identify the prevalence and risk factors of child deaths in Bangladesh. Data were collected from the Bangladesh Demographic and Health Survey, 2011. The results indicate that in Bangladesh there is an association with child deaths and mothers’ age, mothers’ education, social-economic status, birth interval, birth order, baby size and place delivered. For Bangladesh, this study recommends expanding female education to increase mothers’ knowledge, an awareness program about birth order (take one child) and an increase in the birth interval.

## Introduction

Child deaths, which are one of the most important health indicators for a country, represent the socio-economic development of a population and have received considerable attention from national and international agencies in the last few decades because of their inclusion in the Millennium Development Goals (MDGs) [1, 2, 3]. A significant number of projects and programs have been conducted worldwide to reduce the under-five child deaths, especially in resource-limited countries, by two-thirds in 2015. In 2013, WHO reported that among the total of 6.3 million deaths of children aged under-five, 74% of them (4.6 million) died within the infancy period. However, 45% died within the first few months after birth. Almost 83% of this child mortality was due to neonatal, infectious or nutritional conditions. Although the number of under-five deaths has declined worldwide in recent decades (from 12.7 million in 1990 to 6.3 million in 2013), the number is still alarmingly high [4]. In addition, the high burden of child mortality still exists in South Asia (one child dies out of 15 before reaching 5 years of age) as well as Sub-Saharan Africa (one child dies out of 8) [2, 5]. About 33% of global child mortality occurs in South Asian countries, compared to less than 1% in high-income countries [6]. In Bangladesh, the mortality of children has followed a declining trend, having reduced from 133 deaths per 1000 in 1993 to 53 deaths per 1000 in 2011, which confirms that Bangladesh is more likely to reach target 4 of the MDG (48 deaths per 1000 children under 18 years of age) by 2015 [7].

Child deaths, as reported in previous studies, is associated with various socio-demographic, and health related characteristics, e.g., lower parental education, lower socioeconomic status, and higher order of birth are associated with increasing risk of child mortality [8, 9, 10]. Whereas, large birth spacing, lower birth order, urban dwelling, and high socioeconomic status are associated with a lower risk of child mortality [10, 11, 12]. Moreover, another study in Bangladesh identified parent’s education, parent’s occupation, delivery and size of child as significant determinants of child mortality [13, 14]. A few studies also reported a significant difference in child deaths between urban and rural areas [15, 16]. In addition, a multi-country study confirmed that higher national income is associated with a lower rate of child deaths [17].

In developing countries like Bangladesh, women are neglected in almost all aspects of life. In addition, such negligence starts from the childhood, as households, especially from rural areas and among the uneducated have a desire for a male child. Even the family is unhappy for the birth of a female baby. Moreover, social disparity and gender differences for health care exist; for example, girls experience a delay in the intervention for their illness compared to boys [18]. Therefore, the same factor may affect child deaths in a different fashion (severity) for male and female children. Although some studies have been conducted in the field of child mortality in Bangladesh [10, 13, 14, 19], they did not study child deaths separately for male and female children. Therefore, the objective of this study is to determine the risk factors that influence child deaths in Bangladesh among males and females separately.

## Methods

### Sample Design

This is a cross-sectional study using data from the BDHS, 2011.

### Study Setting

There are seven administrative divisions in Bangladesh—Dhaka, Rajshahi, Rangpur, Chittagong, Khulna, Barisal and Sylhet. One division is subdivided into districts (zilas), and each district is divided into administrative units (upazilas), which are further divided into urban and rural areas. An urban area is divided into wards and city corporation units (mohallas) within a ward, while a rural area is divided into union parishes (UP) and villages (mouzas) within a union parish. The 2011 population and housing census, together with the Bangladesh Bureau of Statistics (BBS), were used as the sampling frame for the list of enumeration areas (EAs) in this survey [7].

### Sampling Procedure

A two-stage stratified sample of households was the basis for this survey. In the first stage, 207 clusters in urban areas and 393 in rural areas were selected totaling 600 enumeration areas with proportional probability. In the second stage, on average, 30 households were selected based on the demographic and health survey variables, for both the urban and rural areas in seven divisions by systematic sample. The survey selected 17,842 residential households within this study design [7].

### Main Objective

The main objective of the study is to determine the prevalence and risk factors of child deaths in Bangladesh.

### Data Analysis

The statistical analysis of the results was measured using the IBM statistics Version 21. The χ^2^ test was used to determine the significant associations with the child deaths (boys and girls) and respondents age, place of residence, educational level, socioeconomic status, total number of children ever born, birth interval, mode of delivery and size of child at last birth. Logistic regression analysis was conducted to determine the risk factors with child deaths (boys and girls) and respondents age, place of residence, educational level, socioeconomic status, total number of children ever born, birth interval, mode of delivery and size of child at last birth (Table 1). A P value of 0.05 was considered significant at the 95% CI (Confidence Interval) level. The dependent variable used in the model was the dichotomous binary variable: Y = 1 if have child deaths (boys and girls) and Y = 0, otherwise. Respondents age, place of residence, educational level, socioeconomic status, total number of children ever born, birth interval, mode of delivery and size of child at last birth were considered as predictor variables in this model. Perform preliminary analyses using univariate tests such as chi-square test. Such initial analyses provide a better grasp of what is happening in the data and may point the potential important variable(s) to be used for the multivariate analysis. As a general rule, perform univariate analyses for each independent variable, if they are significant at p-value of 0.25 then select them to the multivariate analysis. If the χ^2^ test for a single independent variable is not significant there is no need to include it in the logistic regression analysis.

**Table 1:** Dependent variables to identify risk factors with child deaths.

Dependent variables	Category
**Sons who have died/Daughters who have died**	0 = No, 1 = Yes
**Independent variables**	
**Respondent’s age**	1 = ≤25, 2 = 26–36 & 3 = ≥37
**Place of residence**	1 = urban and 2 = rural
**Educational level**	1 = no education, 2 = primary education, 3 = secondary education and 4 = higher education
**Socioeconomic status**	1 = Poor, 2 = Middle & 3 = Rich
**Total children ever born**	1 = 1–2, 2 = 3–6 & 3 = 7+
**Birth interval (month)**	1 = <= 24, 2 = 25–48 & 3 = 49+
**Mode of delivery**	0 = Non-Caesarean & 1 = Caesarean
**Size of child at last birth**	1 = Large, 2 = Average & 3 = Small

The rural population refers to the people living in rural areas and urban refers to the people living in urban or city areas. There is a difference between urban and rural in that urban people have access to medical facilities, health care centers, good communication facilities, living standards and schooling compared to rural areas. Poor people refers to those with a monthly income of less than 3000 Bangladeshi Taka, middle class people refers to those with a monthly income of 3000–15,000 and rich people refers to those with a monthly income of more than 15,000 Bangladeshi Taka. If the baby size is 2700–4000 grams (6–9 pounds) that means average baby size, less than 2700 grams is small and more than 4000 grams is large [7].

### Ethics Statement

Ethical approval was obtained from the Ministry of Health and Family Welfare, Dhaka, Bangladesh. International Credit Finance (ICF) provided financial and technical assistance for the survey through USAID/Bangladesh. The BDHS is part of the worldwide Demographic and Health Surveys program, which is designed to collect data on child health [7]. Informed written consent was obtained from all women prior to the study.

## Results

### Distribution of Child Mortality According to Socio-demographic Characteristics

The total women participants included in the study numbered 16,025. The results of the associations of child mortality for several socio-demographic characteristics of women are presented in Table 2. The findings from the present study indicated that the prevalence of male children dying was 13.0% and 11.6% for female children. For both sexes, child deaths was significantly associated with mothers’ age, place of residence, mothers’ educational level, total number of children ever born, socio-economic status of household, preceding birth interval, mode of delivery, and size of child at birth. In observing the reported child mortality from several socio-demographic characteristics, an increasing percentage of child mortality was observed in the case of the mothers’ age. Child mortality was significantly higher in rural areas (14.2% for male child and 12.8% for female child) compared to those in urban areas (10.7% for male child and 9.4% for female child). The mothers’ educational level was significantly associated with child deaths. The child mortality rate for males was higher among women with no formal education (22.0%) followed by primary educated (14.5%), secondary educated (6.3%) and higher educated women (3.4%). A similar pattern of mortality was also observed in the case of female children; however, for each level of mothers’ education, the percentage was comparatively lower than the mortality for males. For both sexes, the reported child mortality was high among poor households (17.4% for males and 14.9% for females) and the percentage decreased with the increasing socio-economic status.

**Table 2:** The χ^2^ test with some selected factors for child deaths, 2011 BDHS.

Variables	Child mortality					
	Boys			Girls		
	Yes	No	χ^2^	Yes	No	χ^2^
Respondent’s age (year)						
**≤25**	244(5.2)	4456(94.8)		190(4.0)	4510(96.0)	
**26–36**	643(10.4)	5520(89.6)	758.38*	571(9.3)	5592(90.7)	774.50*
**≥37**	1194(23.1)	3968(76.9)		1104(21.4)	4058(78.6)	
Place of residence						
**Urban**	593(10.7)	4928(89.3)		521(9.4)	5000(90.6)	
**Rural**	1488(14.2)	9016(85.8)	37.57*	1344(12.8)	9160(87.2)	39.69*
Educational level						
**No education**	980(22.0)	3466(78.0)		933(21.0)	3513(79.0)	
**Primary**	715(14.5)	4214(85.5)	643.28*	614(12.5)	4313(87.5)	689.64*
**Secondary**	346(6.3)	5141(93.7)		284(5.2)	5203(94.8)	
**Higher**	40(3.4)	1123(96.6)		34(2.9)	1129(97.1)	
Socioeconomic status						
**Low**	1008(17.4)	4866(82.8)		877(14.9)	4997(85.1)	
**Middle**	392(12.8)	2681(87.2)	161.64*	389(12.7)	2684(87.3)	134.41*
**High**	681(9.6)	6397(90.4)		599(8.5)	6479(91.5)	
Total children ever born						
**1–2**	241(2.9)	7966(97.1)		191(2.3)	8016(97.7)	
**3–6**	1443(20.3)	5671(79.7)	2242.67*	1296(18.2)	5818(81.8)	2202.10*
**7+**	397(56.4)	307(43.6)		378(53.7)	326(46.3)	
Birth interval (month)						
**<=24**	503(24.2)	1578(75.8)		440(21.1)	1641(78.9)	
**25–48**	819(17.8)	3775(82.2)	161.45*	714(15.5)	3880(84.5)	109.37*
**49+**	702(12.5)	4927(87.5)		663(11.8)	4966(88.2)	
Mode of delivery						
**Caesarean**	70(6.0)	1098(94.0)		55(4.7)	1113(95.3)	
**Non-caesarean**	2011(13.5)	12846(86.5)	54.52*	1810(12.2)	13047(87.8)	58.82*
Child size at last birth						
**Large**	541(9.0)	5499(91.0)		449(7.4)	5591(92.6)	
**Average**	128(10.0)	1154(90.0)	177.76*	146(11.4)	1136(88.6)	177.78*
**Small**	1412(16.2)	7291(83.8)		1270(14.6)	7433(85.4)	

*p<0.05 (level of significance) and *1817 (single women) missing cases were excluded from the analysis

Women with more than six children reported a higher percentage of child mortality for both sexes, whereas it was lower among women with 1–2 children. However, the percentage of child mortality was higher among those children whose preceding birth interval was less than 24 months and lower among those children whose preceding birth interval was more than 48 months (12.5% for males and 11.8% for females). The percentage of child deaths was higher among those children who were delivered normally (13.5% for males and 12.2% for females) than for children who were delivered by caesarean section (6.0% for males and 4.7% for females). The percentage of mortality was also higher among those children whose size at birth was small (16.2% for males and 14.6% for females); however, the percentage was lower among those whose size at birth was large (9.0% for males and 7.4% for females).

The results in table 3 indicated a positive high relationship with the place of residence and socioeconomic status, a negative high correlation with educational level and socioeconomic status, a positive correlation with the total number of children ever born and the mode of delivery and the other variables are negatively or positively correlated with each other.

**Table 3:** Correlation Matrix for child deaths (boys) and socio-demographic variables, 2011 BDHS.

	X_1_	X_2_	X_3_	X_4_	X_5_	X_6_	X_7_	X_8_
**X_1_**	1.00	0.015	0.133	-0.121	-0.358	-0.166	-0.022	-0.530
**X_2_**		1.00	0.004	0.690	-0.050	0.023	0.033	0.024
**X_3_**			1.00	-0.600	0.160	-0.046	-0.116	0.042
**X_4_**				1.00	-0.002	-0.009	-0.087	-0.069
**X_5_**					1.00	0.080	0.590	0.151
**X_6_**						1.00	-0.023	0.169
**X_7_**							1.00	0.225
**X_8_**								1.00

Where, X_1_ = Respondents age,

X_2_ = Place of residence,

X_3_ = Educational level,

X_4_ = Socioeconomic status,

X_5_ = Total number of children ever born,

X_6_ = Birth interval (Month),

X_7_ = Mode of delivery and

X_8_ = Size of child at last birth

From table 4, the results showed an inverse moderate relationship with respondents’ age and total number of children ever born, a positive high relationship with place of residence and socioeconomic status, a positive moderate relationship with the total number of children ever born, and positive moderate relationship with the mode of delivery and total number of children ever born.

**Table 4:** Correlation Matrix with child deaths (girls) and socio-demographic variables, 2011 BDHS.

	X_1_	X_2_	X_3_	X_4_	X_5_	X_6_	X_7_	X_8_
**X_1_**	1.00	0.013	0.133	-0.118	-0.560	-0.161	-0.018	-0.537
**X_2_**		1.00	-0.001	0.680	-0.043	0.020	0.031	0.023
**X_3_**			1.00	-0.354	0.143	-0.044	-0.113	0.038
**X_4_**				1.00	0.009	-0.014	-0.083	-0.070
**X_5_**					1.00	0.096	0.540	0.151
**X_6_**						1.00	-0.024	0.169
**X_7_**							1.00	0.217
**X_8_**								1.00

Where,

X_1_ = Respondents age,

X_2_ = Place of residence,

X_3_ = Educational level,

X_4_ = Socioeconomic status,

X_5_ = Total number of children ever born,

X_6_ = Birth interval (Month),

X_7_ = Mode of delivery and

X_8_ = Size of child at last birth

### Risk Factors of Child Deaths

Table 5 represents the risk factors associated with child deaths. Two separate binary logistic regression models were fitted to identify the socio-economic correlates of child mortality for males and females. The selected socio-demographic variables considered in the two models are mothers’ age, place of residence, mothers’ educational level, total number of children ever born, socio-economic status of household, preceding birth interval, mode of delivery, and size of child at birth. According to the fitted models, all of the selected variables except place of residence and child size at last birth appeared to be statistically significantly correlated to child mortality in the case of males; whereas all of the selected variables except place of residence appeared to be significant correlates of child mortality in the case of females.

**Table 5:** Multiple logistic regression analysis: Risk factors of child deaths in Bangladesh (2011 BDHS).

Variables	Child Deaths					
	Boys			Girls		
	Odd Ratio	df	95%CI	Odd Ratio	df	95%CI
Respondent’s age (year)						
**<=25**	1.00	.	..........	1.00	.	.............
**26–36**	0.670*	1	0.551–0.815	0.736*	1	0.596–0.910
**>=37**	0.983*	1	0.785–1.232	1.123	1	0.883–1.428
Place of residence						
**Urban**	1.00	.	...........	1.00	.	.............
**Rural**	0.924	1	0.815–1.047	0.916	1	0.870–1.133
Educational level						
**No education**	1.00	.	........	1.00	.	..........
**Primary**	0.915	1	0.812–1.032	0.796*	1	0.703–0.902
**Secondary**	0.711*	1	0.606–0.833	0.588*	1	0.497–0.696
**Higher**	0.628*	1	0.437–0.903	0.515*	1	0.346–0.766
Socioeconomic status						
**Low**	1.00	.	.........	1.00	.	.........
**Middle**	0.805*	1	0.698-.928	0.977	1	0.844–1.130
**High**	0.705*	1	0.614-.810	0.806*	1	0.697–0.932
Total children ever born						
**1–2**	1.00	.	........	1.00	.	.........
**3–6**	5.327*	1	4.475–6.341	5.630*	1	4.646–6.824
**7+**	21.421*	1	16.879–27.186	22.338*	1	17.375–28.718
Birth interval (month)						
**<=24**	1.00	.	..........	1.00	.	..........
**25–48**	0.706*	1	0.616–0.808	0.713*	1	0.618–0.823
**49+**	0.551*	1	0.479–0.634	0.622*	1	0.537–0.720
Mode of delivery						
**Caesarean**	1.00	.	............	1.00	.	.......
**Non-caesarean**	1.687*	1	1.253–2.272	1.476*	1	1.066–2.044
Size of child at last birth						
**Large**	1.00	.	............	1.00	.	..........
**Average**	1.017	1	0.810–1.276	1.525*	1	1.221–1.905
**Small**	1.093	1	0.942–1.268	1.068*	1	0.913–1.249
Model Summary:						
Model χ^2^ =	1544.168			1512.039		
Model P value:	0.001			0.001		
-2Loglikelihood =	9456.988			8790.243		
Cox & Snell R^2^ =	0.12			0.12		
Nagelkerke R^2^ =	0.20			0.21		
Overall percentage:	88			87		

*p<0.05 (level of significance) & *1817 (Single women) missing cases were excluded from the analysis

The findings from this study revealed that women who are aged 26–36 years have 33% and 26% lower risk of child mortality for their male children (OR = 0.67, 95%CI = 0.55–0.81) and female children (OR = 0.74, 95%CI = 0.59–0.91), respectively, compared to women aged 25 years and below. There was a negative association between higher education and child mortality. The risk was significantly lower among women who were higher educated followed by secondary and primary educated compared to women with no formal education. For example, in the case of female mortality, the risk was about 50% lower among higher educated women (OR = 0.51, 95% CI = 0.35–0.77) compared to women with no formal education.

Household socio-economic status was an important indicator that was correlated with child deaths between both males and females. Middle class and upper households were 0.81 times (OR = 0.81, 95%CI = 0.69–0.93) and 0.71 times (OR = 0.71, 95%CI = 0.61–0.81) less likely to have sons die, respectively, than poor households; whereas, rich households had 0.81 times less (OR = 0.81, 95% CI = 0.70–0.93) chance of having daughters die. In addition, women whose total number of children ever born was 3–6 and more than 6 were 5.33 times and 21.42 times more likely of having sons die compared to women whose total number of child ever born was one to two. An almost similar risk was seen in the case of girl child’s mortality.

The preceding birth interval also appeared to be another important correlate for both models. The risk of death was 0.71 (OR = 0.71, 95% CI = 0.62–0.81) times less for those whose preceding birth interval was 25–48 months compared to those whose preceding birth interval was less than 25 months. Sons who were delivered by normal section had 1.68 times (OR = 1.68, 95% CI = 1.25–2.27) more risk of mortality compared to sons delivered by caesarean. However, the risk was 1.47 times (OR = 1.47, 95% CI = 1.06–2.04) more in the case of daughters. Daughters whose size at birth was average were 1.53 times (OR = 1.53, 95% CI = 1.22–1.91) more likely to face mortality compared to daughters whose size at birth was large, which indicates that large size at birth is a protective factor for child deaths. For the fitted model, in respect of the Cox and Snell R^2^ and Nagelkerke R^2^, 12% and 20%, respectively, of the variance for boys, and 12% and 21%, respectively, of the variance for girls can be predicted from the linear relationship of the eight independents variables. In respect of the overall percentage, 88% and 87% women were predicted correctly. The overall model was significant when all eight independent variables were entered.

## Discussion

The child death rate reflects a country’s level of socioeconomic improvement and quality of life. It depends on monitoring and evaluating the population and health programs and policies. These rates are also useful in identifying promising directions for the health and nutrition programs in Bangladesh [7]. The results of this study indicate that for the death of children less than 18 years of age by gender difference, a child’s death depends on mothers’ age, mothers’ education, socioeconomic status, geographic difference and mass media awareness program. Births to younger women can generally be identified as high-risk births [2, 20]. This study confirms that children born (especially boy) to women of more than 25 years of age are at lower risk of child deaths. In Bangladesh, majority younger women are not developed economically, emotionally, physically, and are less mature, which, together with other aspects of their life, have a detrimental impact on their children.

Women’s education has an inverse relation with children’s deaths. Educational level is highly association with lower mortality risks because women’s education provides the information about better pregnancy and child health care [7, 21]. The results show that the child deaths for boys is 0.711 times lower risk for those whose mothers have completed higher education than for those whose mothers are illiterate (6.3% to 22.0% deaths), and for the child deaths of girls it is 0.588 times lower for those whose mothers have completed higher education than for those whose mothers are illiterate (5.2% to 21.0% deaths). Mothers with higher education reduce the risk of child deaths by about half. Educated women have better income, higher health literacy and power to make healthier decisions for their health and that of their children [3].

Child deaths in urban and rural areas in Bangladesh have a statistically significant risk difference. Some studies have identified that child mortality in rural areas is at higher risk than in urban areas [3, 10–14, 15, 22]. This study shows that there is a significant association with urban-rural and gender difference, such as the deaths of urban boys and girls (10.7% to 9.4%) and that of rural boys and girls (14.2% to 12.8%). This is because urban areas have good access to basic medical facilities and health care compared to rural areas.

The wealth index of women is the main way to determine the health status in a country. Some studies have shown a positive statistical association between low income and child deaths but an opposite relationship between high income and child deaths [3, 8, 17]. Similarly, in this study the lower income women are at greater risk for child deaths (boys and girls). Because lower income women have access to fewer medical facilities in rural areas, such as hospitals, doctors, paramedics or community based health workers.

Birth order is an essential measure of child deaths. Although some previous studies have shown that higher ranked birth order present a higher risk of child deaths, a few other studies have indicated that lower ranked birth order experience an increased risk of child deaths. The infants of first birth have a higher risk of neonatal mortality than fourth or higher-ranked births in India [23, 24, 25]. A study conducted in Taiwan [26] showed that children with first and fifth-ranked births are at higher risk of early child deaths, with same in Nigeria [27]. In this study, women who have children ever born 3–6 is 5.33 times more risk of boys child death, and children ever born more than 7 is 21.42 times more risk of sons child death as compared to women who had at least one child ever born. An almost similar risk is seen in the case of girl child deaths. Short birth interval and child deaths have a significant association in Bangladesh [3, 28]. Another study also found a similar risk association [29]. The risk of death is 0.71 times less for those women having a birth interval of 25–48 months compared to those women having a birth interval of less than 25 months. One of the limitations in this study is that the information was self-reported.

## Conclusion

The causes of child deaths is important for health sector planning, especially in determining the program needs, and monitoring for improving intervention and reassessment of health priorities. Increasing mothers’ education and making them productive to improve their income are important aspects for reducing child deaths. Hence, it is having needed to increase rural based community education programs about child deaths. Being a younger mother at birth, short birth interval and birth order have been identified as risk factors for increased child deaths in Bangladesh. Interventions targeted at empowering women and much effort in emerging rural areas is necessary. Reducing young motherhood and increasing satisfactory birth spacing are also necessary to reduce child deaths in Bangladesh.

## Supporting Information

S1 Checklist STROBE checklist. (DOC)
